# Methotrexate Promotes Platelet Apoptosis via JNK-Mediated Mitochondrial Damage: Alleviation by *N*-Acetylcysteine and *N*-Acetylcysteine Amide

**DOI:** 10.1371/journal.pone.0127558

**Published:** 2015-06-17

**Authors:** Manoj Paul, Mahadevappa Hemshekhar, Ram M. Thushara, Mahalingam S. Sundaram, Somanathapura K. NaveenKumar, Shivanna Naveen, Sannaningaiah Devaraja, Kumar Somyajit, Robert West, Basappa, Siddaiah C. Nayaka, Uzma I. Zakai, Ganesh Nagaraju, Kanchugarakoppal S. Rangappa, Kempaiah Kemparaju, Kesturu S. Girish

**Affiliations:** 1 Department of studies in Biochemistry, University of Mysore, Manasagangothri, Mysore, 570 006, India; 2 Department of Internal Medicine, Manitoba Centre for Proteomics and Systems Biology, University of Manitoba, Winnipeg, R3E3P4, Canada; 3 Applied Nutrition Discipline, Defence Food Research Laboratory, Mysore, 570 011, India; 4 Department of Studies and Research in Biochemistry, Tumkur University, Tumkur, 572 103, India; 5 Department of Biochemistry, Indian Institute of Science, Bangalore, 560 012, India; 6 Organosilicon Research Center, University of Wisconsin, Madison, WI, 53706–1396, United States of America; 7 Laboratory of Chemical Biology, Department of Chemistry, Bangalore University, Bangalore, India; 8 Department of Applied Botany and Biotechnology, University of Mysore, Manasagangothri, Mysore, 570 006, India; 9 Department of Studies in Chemistry, University of Mysore, Manasagangothri, Mysore, 570 006, India; University of Windsor, CANADA

## Abstract

Thrombocytopenia in methotrexate (MTX)-treated cancer and rheumatoid arthritis (RA) patients connotes the interference of MTX with platelets. Hence, it seemed appealing to appraise the effect of MTX on platelets. Thereby, the mechanism of action of MTX on platelets was dissected. MTX (10 μM) induced activation of pro-apoptotic proteins Bid, Bax and Bad through JNK phosphorylation leading to ΔΨ*m* dissipation, cytochrome c release and caspase activation, culminating in apoptosis. The use of specific inhibitor for JNK abrogates the MTX-induced activation of pro-apoptotic proteins and downstream events confirming JNK phosphorylation by MTX as a key event. We also demonstrate that platelet mitochondria as prime sources of ROS which plays a central role in MTX-induced apoptosis. Further, MTX induces oxidative stress by altering the levels of ROS and glutathione cycle. In parallel, the clinically approved thiol antioxidant *N*-acetylcysteine (NAC) and its derivative *N*-acetylcysteine amide (NACA) proficiently alleviate MTX-induced platelet apoptosis and oxidative damage. These findings underpin the dearth of research on interference of therapeutic drugs with platelets, despite their importance in human health and disease. Therefore, the use of antioxidants as supplementary therapy seems to be a safe bet in pathologies associated with altered platelet functions.

## Introduction

Methotrexate (MTX) is a well-known anti-cancer and disease modifying anti-rheumatic drug, actively used in the treatment of a wide spectrum of human ailments including cancer, rheumatoid arthritis (RA), psoriasis, asthma, primary biliary cirrhosis, and is also as an immunosuppressant [1–4]. However, several studies have demonstrated the adverse effects of MTX in long term or high dose treatment; one of the major concerns being its pro-oxidant and non-specific action [5]. The average MTX levels in cerebrospinal fluid and serum is reported to be 17.1 μM and 779 μM respectively in various cancer patients treated with high-dose MTX [6]. Of late, Conway et al. [7] conducted a meta-analysis study involving randomized controlled trials and demonstrated a significant correlation in the risk of lung disease in MTX-treated RA patients. Another recent retrospective review describes the incidences of MTX withdrawal in RA patients due to unacceptable adverse effects. The study claims that about 32.7% patients experienced gastrointestinal problems, while 20.6% had nausea. Several cancer and RA patients also experienced significant respiratory, hepatic and renal dysfunctionalities [8]. The MTX-induced organ damage is attributed to oxidative stress due to excessive production of reactive oxygen/nitrogen species (ROS/RNS) [9]. The circulatory MTX is shown to induce acute and chronic augmentation in plasma homocysteine levels, which in turn is responsible for elevated levels of ROS. It also induces depletion of glutathione (GSH) by diminishing the intracellular NADPH levels and thus, altering redox homeostasis [10].

Lately, anemia and thrombocytopenia have been linked with MTX overdose or on prolonged treatment. Many studies report a significant case of thrombocytopenia among MTX-treated RA and cancer patients [8], [11]. Thus, it is highly interesting to evaluate the mechanism of action of MTX on platelets and their functions. Though the cytotoxic nature of MTX is known, its effect on highly sensitive platelets is not clear. In addition, the role played by microparticles (MPs) (that are generated by activated and/or apoptotic platelets) in the propagation of RA is well-known. Boilard et al. [12] have demonstrated the presence of elevated levels of platelet-derived MPs in the synovial fluid from RA patients. Platelet-derived MPs were shown to have a pro-inflammatory effect on synovial fibroblasts [12]. Despite the fact that platelets are anuclear, they undergo apoptosis either naturally or upon external stimulation [13], [14]. Hitherto, several studies have demonstrated the mitochondria-mediated classical apoptotic events. The elevated plasma levels of ROS are among the key factors responsible for premature apoptosis of platelets [15], [16]. They are known to alter mitochondrial membrane potential (∆ψ*m*) and trigger cytochrome c (cyt. c) release, which would activate caspases and communicate phagocytic signals via phosphatidylserine (PS) externalization [17], [18]. Thus, MTX-induced ROS generation may play a key role in platelet physiology and functions in RA patients. Besides, studies have also reported the use of natural or synthetic small molecules to prevent adverse effects of MTX. Many reports including our own have shown the platelet protective actions of small molecules [17], [19]. Hence, this study for the first time makes an attempt to uncover the mechanistic action of MTX on platelet functions and survivability. We examined the link between MTX-induced platelet apoptosis and JNK-mediated mitochondrial damage. We also demonstrated the counter action of *N*-acetylcysteine (NAC) and *N*-acetylcysteine amide (NACA) on MTX-induced platelet apoptosis. Though NAC is a potent antioxidant, its low bioavailability lead to the synthesis of NACA, which is more efficient than NAC in all aspects including bioavailability [20].

## Materials and Methods

### Chemicals/ Reagents

Calcium ionophore (A23187), NAC, NACA, leupeptin hydrochoride, 10-nonyl acridine orange (NAO), dicumarol [3,3´-Methylene-bis(4-hydroxy-coumarin)], Mito-TEMPO, epinephrine, adenosine diphosphate (ADP), monoclonal phosphotyrosine antibody (clone PT-66), dithiotritol, and all fluorescent dyes required to determine platelet apoptosis were from Sigma Chemicals, USA. Cyt. c antibody was from Epitomics, USA. z-DEVD-fmk, Caspase-3, Caspase-9, Caspase-8, Bad, Bax, Bcl-2 and tBid antibodies were from Santa Cruz Biotechnology, Inc. USA. COX IV, β-Tubulin, phospho-eIF2α (ser51), eIF2α, phospho-JNK 1/2 (Thr183/Tyr185) and JNK 1/2 antibodies were from Cell Signaling and Technologies, Inc. USA. Collagen type-I was from Chrono-log Corporation, USA. 3-(4,5-dimethylthiazol-2-yl)-2,5-diphenyltetrazolium bromide (MTT) was from HiMedia Laboratories, India. Lactate Dehydrogenase kit was from Agappe diagnostics Ltd., India. Methotrexate was from Sisco Research Laboratories Pvt. Ltd., India. All other reagents were of analytical grade.

### Preparation of platelet-rich plasma and washed platelets

Venous blood was drawn from healthy, non-smoking, drug-free human volunteers with written consent and was approved by Institutional Human Ethical Committee (IHEC-UOM No. 40Res/2013-14), University of Mysore, Mysore. All methods were in accordance with IHEC guidelines and the study was approved by IHEC (IHEC-UOM No. 40Res/2013-14), University of Mysore, Mysore. The drawn blood was immediately mixed with acid citrate dextrose (ACD) anticoagulant and processed as described previously to obtain platelet rich plasma (PRP) and washed platelets [21]. The cell count was determined in both PRP and washed platelet suspensions using a Neubauer chamber and adjusted to 5×10^8^ cells/mL in the final suspension using platelet poor plasma/Tyrode’s albumin buffer (pH 7.4).

### Determination of platelet apoptotic markers

Platelet apoptotic markers like increase in ROS, intracellular calcium, peroxidation of cardiolipin and PS externailization in platelets were determined as described previously using Varioskan multimode plate reader (Thermo Scientifics, USA). Briefly, PRP and washed platelet suspensions (5×10^6^ cells/mL) were taken separately and treated with A23187 (10 μM) as positive control or MTX in increasing doses (0–50 μM) and the final volume was made up to 200 μL with HEPES-buffered saline (HBS, pH 7.45) and incubated at 37°C for 1 h. For inhibition studies, platelets were treated with MTX (50 μM) in presence or absence of Dicumarol/ z-DEVD-fmk/ Mito-TEMPO/ NAC/ NACA at different doses (0–1000 μM). The control (untreated) and treated platelets were then incubated with respective fluorescent probes. Finally, cells were collected by centrifugation and fluorescence was recorded as described previously [19], [22].

### Flow cytometry

For flow cytometry analysis washed platelets (1×10^6^ cells/mL) were stimulated with A23187 (10 μM) as positive control or MTX in increasing doses (0–50 μM) and incubated at 37°C for 1 h. For inhibition studies, platelets were treated with MTX (50 μM) in presence or absence of NAC/NACA at different doses (0–1000 μM). After incubation, cells were stained using CM-H2DCFDA, JC-1 and Annexin V-FITC, washed and analyzed by FACSVerse^TM^ flow cytometer (BD Biosciences, USA) [23–25].

### Western blot analysis

Washed platelets (1×10^8^ cells/mL) were stimulated independently with MTX (0–50 μM) and for inhibition studies, MTX treated platelets were incubated with different concentrations of Mito-TEMPO/ Dicumarol/ z-DEVD-fmk/ NAC/ NACA. The samples were then incubated for 30 min to determine activation/expression levels of tyrosine phosphorylated proteins, phospho-eIF2-α, eIF2-α, Bad, Bax, Bcl-2, tBid and caspase-8, 1 h for cyt. c, caspase-9 and -3 activation and 0–4 h for phospho-JNK1/2 and JNK1/2. Further, platelet suspensions were lysed by adding 10 μL of HEPES buffer containing 2% SDS, 5 mM *N*-ethylmaleimide, 5 mM Na_3_VO_4_, 10 mM EDTA and 10 mM PMSF. Following centrifugation, the supernatants were separated on SDS-PAGE and electroblotted on to a PVDF membrane (MDI membranes, Advanced Microdevices Pvt Ltd, India). Membrane was cut based on the molecular weight and blocked using 5% BSA. Further, the blots were probed independently with respective antibodies, developed by enhanced chemiluminescence method [19] and visualized using western blot chemiluminescence imaging system (Alliance 2.1, Uvitec, UK).

### Electron transport chain (ETC) assays

Assays for ETC complexes I, II, III and IV were performed as described accordingly [26]. Briefly, platelet mitochondrial fractions were treated with MTX in increasing doses (0–100 μM) and for inhibition studies, platelets treated with MTX were incubated with different doses of NAC/NACA (0–1000 μM) for 30 min at 37°C. Complex I activity was expressed as mM NADH oxidized/min/mg protein, Complex II activity was expressed as mM dichlorophenolindophenol (DCIP) reduced/min/mg protein, Complex III activity was expressed as mM cyt. c reduced/min/mg protein and Complex IV activity was expressed as first-order rate constant (k) of mM cyt. c oxidized/min/mg protein.

### Measurement of γ-glutamyltransferase (GGT) activity

GGT activity in washed platelets was determined as described accordingly [27]. The results were calculated using molar extinction coefficient of *p*-nitroanilide (9,900 M^-1^cm^-1^) at 405 nm and expressed as mM *p*-nitroanilide formed/min/mg protein.

### Microparticle isolation from platelets

Washed platelets (5×10^8^ cells/mL) were treated independently with A23187 (10 μM) or MTX in increasing doses (0–50 μM) and for inhibition studies, pre-loaded platelets with MTX (50 μM) were incubated with different doses of NAC/NACA (0–1000 μM) for 1 h at 37°C. After incubation, samples were gently mixed and to separate microparticles (MPs), samples were centrifuged at 14,200×*g* for 15 min at 4°C and the supernatant thus obtained was considered as MP-rich fraction [28]. Supernatants were used to determine coagulant activity.

### Coagulant activity

The plasma coagulation property was determined according to the method of Condrea et al. [29]. Normal human citrated plasma (200 μL) was incubated with different doses of MTX (0–50 μM) and MP-rich fraction (20 μL), and the clotting time was recorded against a light source.

### Platelet aggregation

Platelet aggregation was determined by turbidimetric method with a dual channel Chrono-log model 700–2 aggregometer (Havertown, USA). Briefly, 250 μL of PRP was taken in siliconized glass cuvette and pre-incubated for 3 min at 37°C with different concentrations of MTX (0–50 μM), and the aggregation was initiated by the addition of collagen (2 μg/mL)/ADP (5 μM)/epinephrine (10 μM). The aggregation was then followed with constant stirring at 1200 rpm for 6 min at 37°C [19].

### MTT assay

MTT colorimetric assay was performed to assess the cell viability. PRP was treated independantly with A23187 (10 μM) or different doses of MTX (0–50 μM). For inhibition studies, platelets treated with MTX (50 μM) were incubated with different concentrations of NAC/NACA (0–1000 μM). After 1 h of incubation 250 μM of MTT was added and incubated for additional 3 h. Thereafter, MTT was removed and the remaining formazan crystals was completely dissolved in DMSO. The absorbance at 570 nm was recorded using multimode plate reader [15].

### Measurement of LDH leakage

PRP was treated as described in the above section and platelets were pelleted by centrifuging at 1,700×*g* for 10 min. Supernatants were used to detect LDH release by using Agappe LDH kit, according to the manufacturer’s protocol. The assay was performed in a time course of decrease in NADH absorbance at 340 nm for 3 min using spectrophotometer (Beckman Coulter DU-730, CA, USA) [15].

### Protein estimation

Protein estimation was carried out according to the method of Lowry et al. [30] using BSA as standard.

### Statistical analysis

Results were expressed as mean ± SEM of five independent experiments. Statistical significance among groups was determined by one way analysis of variance (ANOVA) followed by Tukey’s test for comparison of means [n = 5, p*/^#^< 0.05, p**/^##^< 0.01, p***/^###^< 0.001; *: significant compared to control. #: significant compared to MTX alone treated group].

## Results

### MTX-induced ROS generation and its inhibition by NAC/NACA

**Fig 1:**
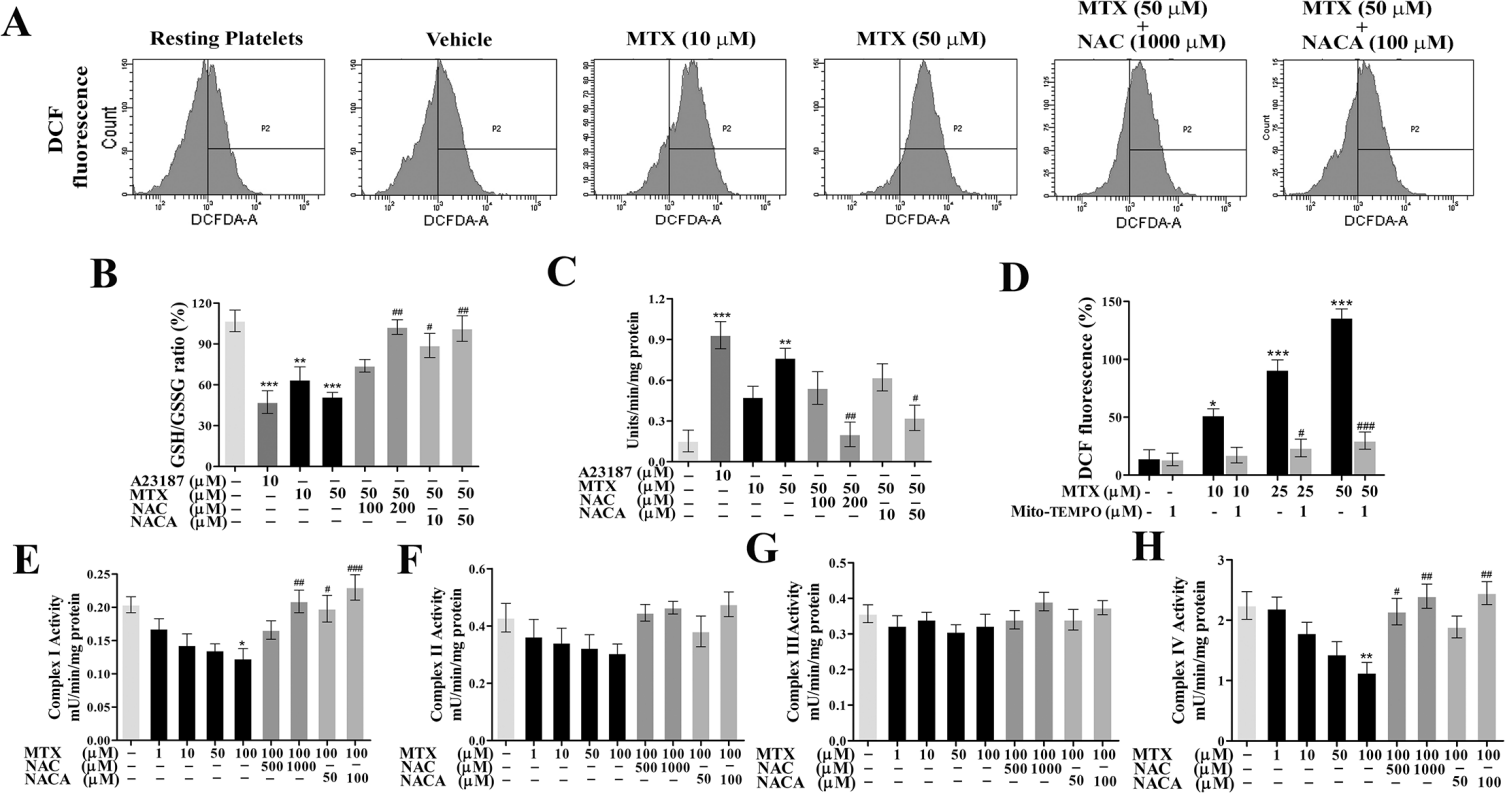
MTX altered ROS levels in platelets and its inhibition by NAC/NACA/Mito-TEMPO. **A,** FACS analysis of ROS generation in washed platelets treated with MTX in presence or absence of NAC/NACA. **B,** GSH/GSSG ratio and (**C**) γ‑Glutamyltransferase activity. **D,** Effect of Mito-TEMPO on MTX induced ROS generation, expressed as percentage increase in DCF fluorescence. Effect of MTX in presence or absence of NAC/NACA on components of mitochondrial electron transport chain, (**E**) Complex I-NADH: ubiquinone oxidoreductase activity (**F**) Complex II-succinate: ubiquinone oxidoreductase activity (**G**) Complex III-coenzyme Q: cytochrome c-oxidoreductase activity and (**H**) Complex IV-cytochrome c oxidase activity. Values are presented as mean ± SEM (n = 5). p*/#< 0.05, p**/##< 0.01, p***/###< 0.001; *: significant compared to control. #: significant compared to MTX.

In order to understand whether MTX has any influence on platelet functions, initially platelets treated with MTX were evaluated for endogenous generation of ROS. FACS analysis of MTX-treated platelets showed significant increase in endogenous generation of ROS compared to control (Fig 1A). Further, MTX displayed significant decrease in GSH/GSSG levels when compared to control pointing out the oxidative stress in platelets (Fig 1B). GGT, whose upsurge is a mark of cellular stress, was also found to be elevated in MTX-treated platelets (Fig 1C). However, no significant alteration in glutathione peroxidase and reductase activities was observed (data not shown). Interestingly, treatment with NAC and NACA significantly abolished MTX-induced ROS generation, restored GSH/GSSG levels and GGT activity (Fig 1A–1C).

Mito-TEMPO, a mitochondria-targeted ROS antagonist completely abolished MTX-induced ROS production indicating that mitochondria are the major sources for ROS production in platelets (Fig 1D). Therefore, effects of MTX on mitochondrial components of ETC were assessed. MTX at 100 μM concentration reduced NADH: ubiquinone oxidoreductase (Complex-I) activity up to 42% (Fig 1E) whereas, succinate: ubiquinone oxidoreductase (Complex-II) activity was slightly reduced (Fig 1F). Furthermore, coenzyme Q: cytochrome c oxidoreductase (Complex-III) activity remained unaffected (Fig 1G); while there was a significant reduction in the activity of cyt. c oxidase (Complex-IV) up to 50% upon exposure to 100 μM MTX (Fig 1H). Surprisingly, the presence of NAC and NACA significantly restored the activities of complexes I and IV of ETC, confirming their protective role against MTX-induced toxicities on platelets (Fig 1E and 1H).

### MTX-induced mitochondrial dysfunction and its protection by NAC/NACA

**Fig 2:**
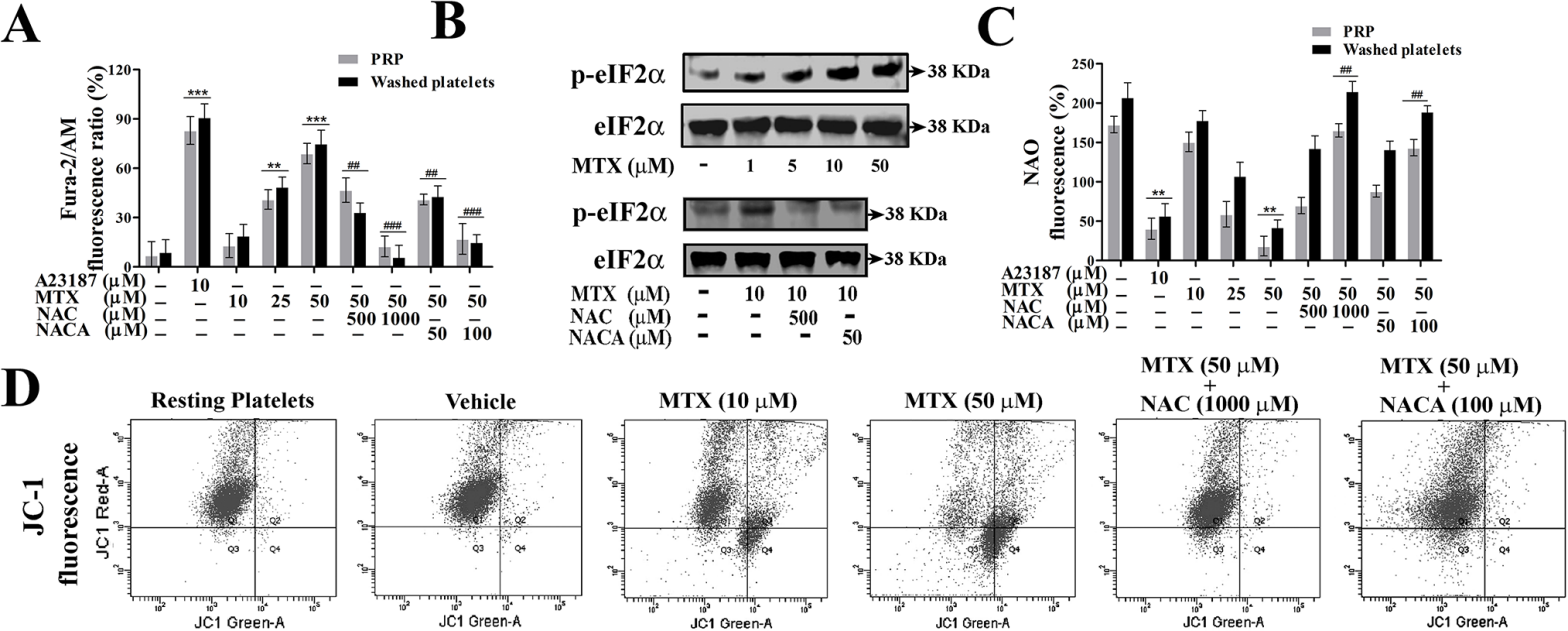
MTX altered ER stress and mitochondrial apoptotic markers and its inhibition by NAC/NACA. Effect of MTX in presence or absence of NAC/NACA on (**A**) changes in intracellular calcium levels, (**B**) phospho-eIF2-α expression and (**C**) peroxidation of cardiolipin, (**D**) FACS analysis of ΔΨ*m* depolarization in washed platelets treated with MTX in presence or absence of NAC/NACA. Values are presented as mean ± SEM (n = 5), expressed as percentage increase in (**A**) fura-2/AM and (**C**) NAO fluorescence. p*/#< 0.05, p**/##< 0.01, p***/###< 0.001; *: significant compared to control. #: significant compared to MTX. Membrane was cut based on the molecular weight, probed with antibody of interest and band of interest were presented.

When cellular oxidative stress exceeds beyond its control the cells are destined to undergo apoptosis, which includes Ca^2+^ion release, altered ΔΨ*m*, cyt. c release from mitochondria and downstream apoptotic events. MTX significantly induced the release of Ca^2+^ from endoplasmic reticulum (ER) (Fig 2A) and immunoblots of eIF2-α suggest that platelets were under ER stress during MTX treatment (Fig 2B).

Altered ROS and intracellular calcium levels cause mitochondrial transition including dissipation of ΔΨ*m* and peroxidation of cardiolipin. It was observed that MTX dose-dependently increased peroxidation of cardiolipin (Fig 2C) and dissipated ΔΨ*m* (Fig 2D). Whereas, treatment with NAC/NACA significantly restored Ca^2+^ levels, ER stress, cardiolipin peroxidation, and ΔΨ*m* (Fig 2A–2D).

### MTX induces platelet apoptosis; protection by NAC/NACA

**Fig 3:**
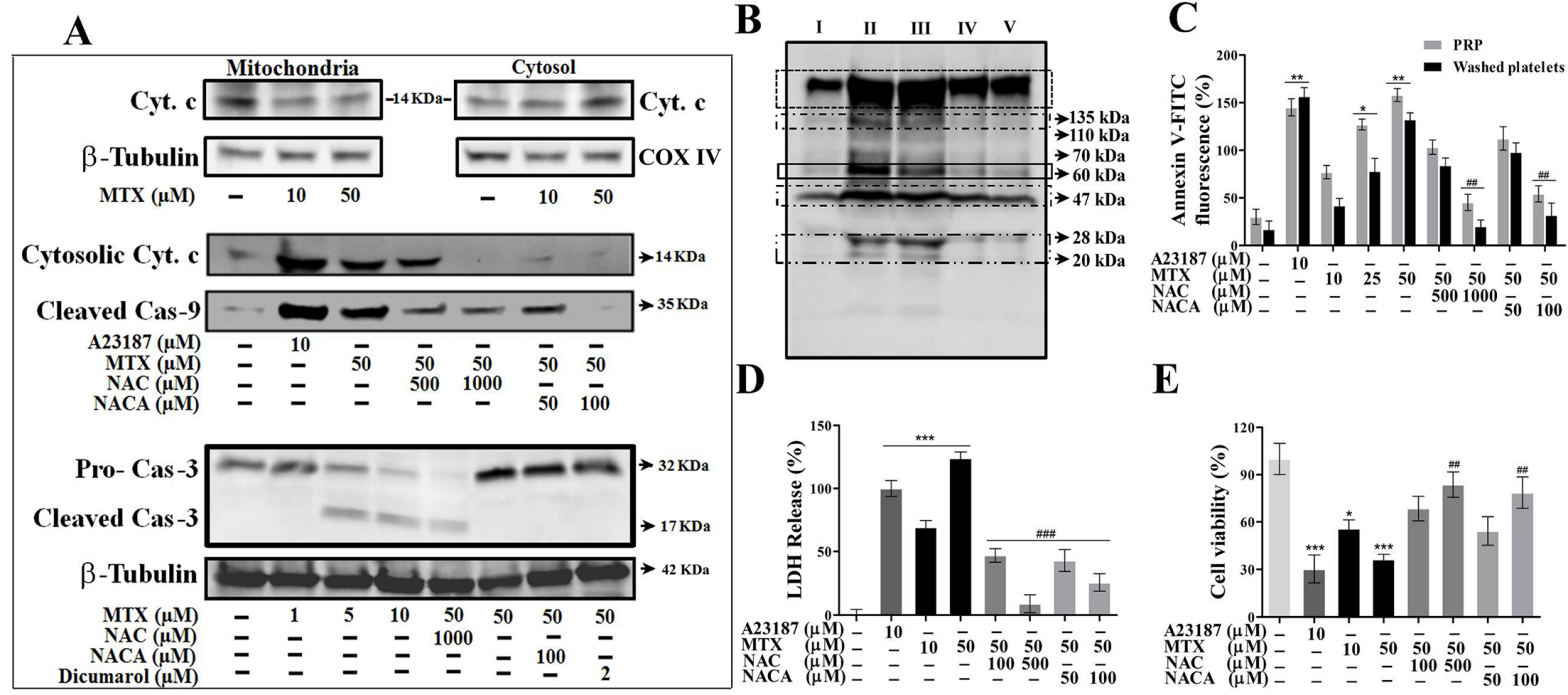
MTX induced platelet apoptosis and its inhibition by NAC/NACA. Effect of MTX in presence or absence of NAC/NACA/Dicumarol on (**A**) cyt. c release from mitochondria to cytosol, activation of caspase-9 and caspase-3, (**B**) protein tyrosine phosphorylation, (**C**) PS externalization, (**D**) LDH release and (**E**) MTT cell viability assay. **B,** Lane I: resting platelets (untreated), Lane II: platelets treated with A23187 (10 μM), Lane III: platelets treated with MTX (50 μM), Lane IV: pre-loaded platelets with MTX (50 μM) and incubated with NAC (500 μM), Lane V: pre-loaded platelets with MTX (50 μM) and incubated with NACA (50 μM). Values are presented as mean ± SEM (n = 5), expressed as percentage increase in (**C**) Annexin V-FITC fluorescence. COX IV and β-Tubulin were used as loading control. p*/#< 0.05, p**/##< 0.01, p***/###< 0.001; *: significant compared to control. #: significant compared to MTX. Membrane was cut based on the molecular weight, probed with antibody of interest and band of interest were presented.

Peroxidized cardiolipin aids in the formation of mitochondrial permeability transition pore (MPTP) through which cyt. c escapes into cytosol, which in turn induces caspase-3 activation and finally PS externalization. MTX was found to induce cyt. c release from mitochondria to cytosol, expression of cleaved caspases, particularly caspase-9 and -3 (Fig 3A). Recently it has been demonstrated that activated caspases cleave variety of cellular substrates and thus expose new sites on proteins for phosphorylation [31]. Supportively, platelets treated with MTX comprised of increased tyrosine-phosphorylated protein content (Fig 3B). MTT also significantly induced PS scrambling, the final hallmark of apoptosis (Fig 3C). Interestingly, treatment with NAC/NACA abrogated cyt. c release, caspase-9 and -3 activation, and PS externalization (Fig 3A–3C). Further, LDH release and MTT assays confirmed the cytotoxic effect of MTX on platelets, whereas NAC/NACA proved to be protective against MTX-induced cytotoxic properties (Fig 3D and 3E). Besides, MTX treatment did not affect platelet aggregation stimulated by collagen/ADP/epinephrine (S1 Fig).

### MTX induces platelet apoptosis via JNK activation

**Fig 4:**
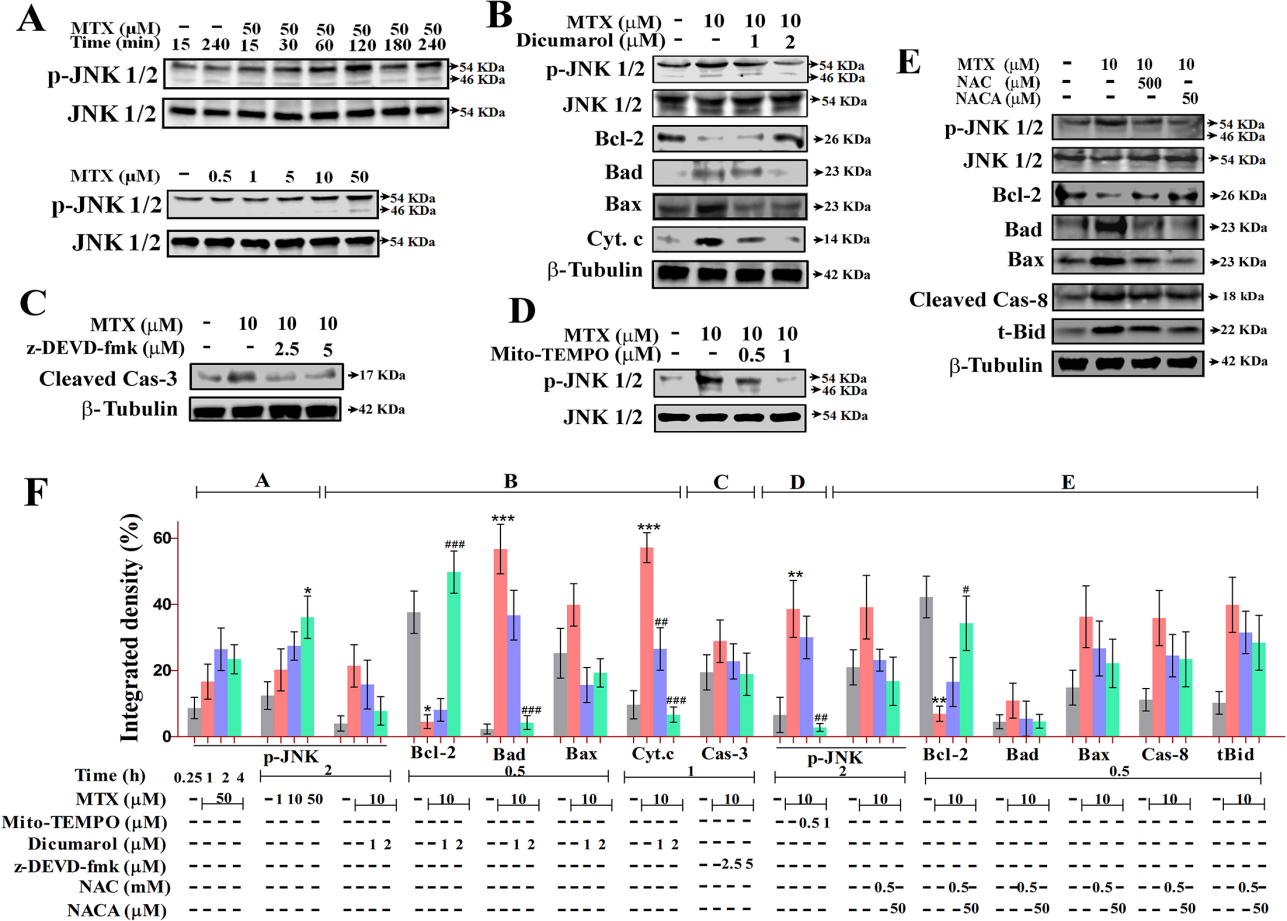
JNK activation by mitochondrial ROS in MTX-treated platelets and its reversal by NAC/NACA. **A,** Immunoblot showing the expression of phospho-JNK in time- and concentration-dependent manner in MTX-treated platelets. **B**, Immunoblots showing the effect of Dicumarol on the expression levels of phospho-JNK, Bcl-2, Bad, Bax, cyt. c, Cas-3 in MTX treated platelets. **C,** Immunoblots showing the effect of z-DEVD-fmk on the expression level of Cas-3 in MTX-treated platelets. **D,** Immunoblots showing the effect of Mito-TEMPO on the expression levels of phospho-JNK in MTX-treated platelets. **E,** Effect of NAC/NACA on the expression levels of phospho-JNK, Bcl-2, Bad, Bax, tBid and Cas-8 in MTX-treated platelets. **F,** Representative densitogram of immunoblots present in panel **A, B, C, D,** and **E**. JNK and β-Tubulin were used as loading control. Membrane was cut based on the molecular weight, probed with antibody of interest and band of interest were presented.

In order to dissect the molecular mechanism through which MTX induces platelet apoptosis, various apoptotic signaling pathways were probed. MTX-treated platelets exhibited time dependent and concentration-dependent increase in phosphorylated form of JNK1/2. (Fig 4A). The phosphorylated form of JNK activates Bad, Bax and inhibits Bcl-2, contributing towards mitochondrial dysfunction. However, Dicumarol a JNK specific inhibitor significantly inhibited JNK phosphorylation suggesting it to be a key event. MTX-treated platelets also showed increased expression of Bad, Bax, and suppression of anti-apoptotic Bcl-2 proteins, which was also reversed in presence of Dicumarol (Fig 4B).

Moreover, Dicumarol and z-DEVD-fmk also inhibited MTX-induced caspase-3 activation suggesting that caspase-3 activation might be mediated by upstream JNK phosphorylation. (Fig 4B and 4C). Mito-TEMPO, a specific mitochondrial ROS quencher significantly abolished MTX-induced phosphorylation of JNK confirming that JNK activation was primed by mitochondrial ROS (Fig 4D). Additionally, NAC/NACA significantly decreased JNK activation, restored Bax, Bad, tBid, Bcl-2 and Cas-8 levels in MTX-treated platelets (Fig 4E).

**Fig 5:**
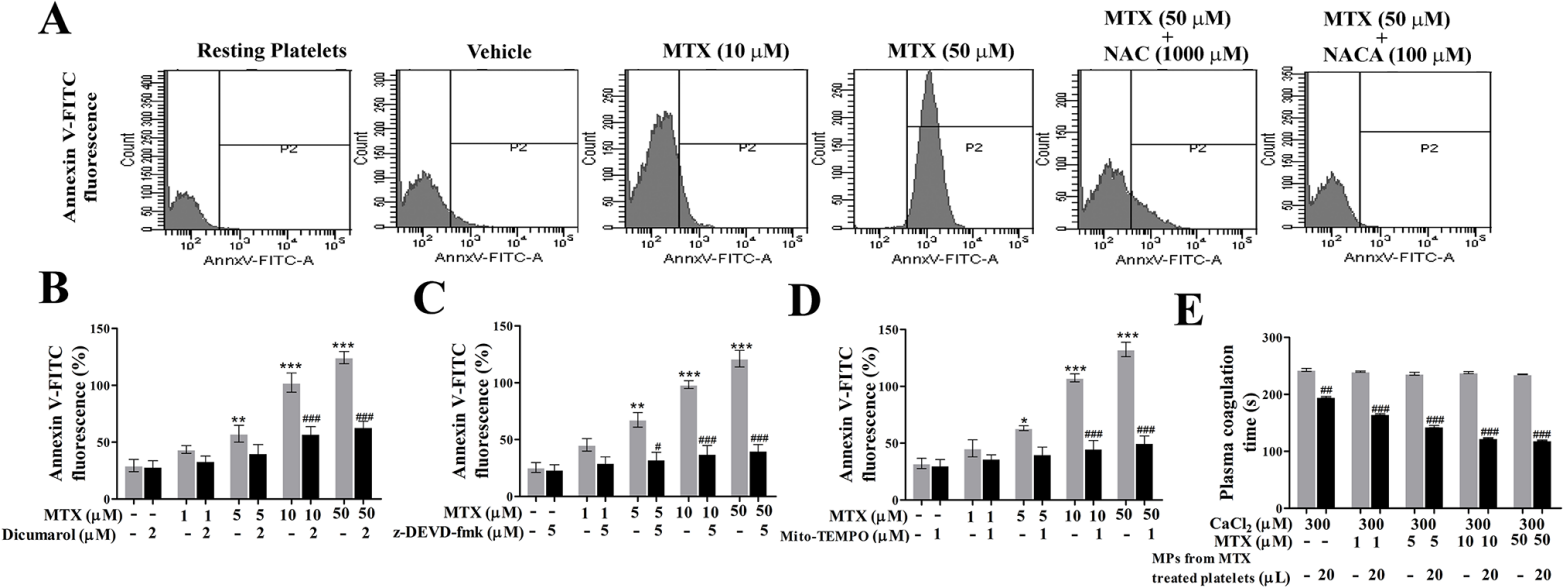
A, Effect of NAC/NACA on MTX-induced PS externalization. Effect of (**B**) Dicumarol (**C**) z-DEVD-fmk and (**D**) Mito-TEMPO on MTX induced PS externalization on washed platelets. (**E**) Effect of microparticle-rich fraction on plasma clotting time obtained from MTX-treated platelets. Values are presented as mean ± SEM (n = 5), expressed as percentage increase in (**B-D**) Annexin V-FITC fluorescence. p*/#< 0.05, p**/##< 0.01, p***/###< 0.001; *: significant compared to control. #: significant compared to MTX.

Finally, FACS data confirmed the protective effects of NAC/NACA in reducing PS externalization in MTX-activated platelets (Fig 5A). MTX-mediated PS externalization was also performed in the presence of Mito-TEMPO, Dicumarol and z-DEVD-fmk. PS externalization was significantly obliterated in the presence of Mito-TEMPO, Dicumarol and z-DEVD-fmk suggesting, MTX-induced platelet apoptosis is driven by mitochondrial ROS-mediated JNK activation (Fig 5B–5D).

Besides, MTX did not alter plasma clotting time whereas, MP-rich fraction obtained from MTX-treated platelets were able to significantly decrease plasma clotting time (Fig 5E). Altogether, these findings suggest that MTX induces platelets apoptosis via JNK activation, and treatment with NAC/NACA appeared to be promising in confronting MTX-induced platelet apoptosis.

## Discussion

MTX is an important therapeutic drug for a variety of human malignancies and autoimmune disorders [32–34]. However, MTX is reported to repress the cellular antioxidant system, thereby augmenting oxidative stress in many organs such as liver, kidney, central nervous system and small intestine [35]. MTX is demonstrated to increase the amount of H_2_O_2_ and stimulate neutrophils, leading to the release of other free radicals causing cellular damage [36]. It reportedly hinders the remethylation of homocysteine resulting in elevated levels of homocysteine, causing ROS generation including H_2_O_2_ [37]. The elevated ROS play a key role in arbitrating apoptosis and cellular signaling pathways, which have been held responsible for MTX-induced tissue damage [38]. Several RA and cancer patients treated with MTX are diagnosed with significant respiratory and renal dysfunctionalities, neutropenia and thrombocytopenia [39], [40]. Here we investigate the effect of MTX on anuclear platelets to understand whether MTX can induce toxicity in platelets as they do in other cells or activate key signaling cascade as an explanation to MTX-induced thrombocytopenia. We also demonstrate the mitigation of MTX-induced platelet apoptosis and toxicities by potent antioxidants NAC/NACA, emphasizing the use of antioxidants as supplementary therapy in MTX treatment.

The hepatic and renal toxicities observed during MTX treatment, are deemed to be the result of oxidative stress due to reduction in GSH content [41]. GGT cleaves GSH to glutamic acid and cysteinylglycine and thus augments the levels of GGT in serum, an essential biochemical marker of early oxidative stress, in the joints, synovial fluid and spleen of MTX-treated RA patients [42]. In the present study, although MTX did not augment the activities of glutathione peroxidase and glutathione reductase, it was found to decrease the GSH/GSSG ratio and increase GGT activity in platelets. This observation is particularly crucial in regard to the use of MTX in RA patients, considering the key role played by activated platelets and platelet-derived MPs in the pathophysiology of RA. NAC and NACA were able to restore the GSH/GSSG ratio to normal and also impair the heightened activity of GGT; thus proving to be effective in preventing MTX-induced oxidative stress at various stages.

Studies have demonstrated that MTX induces intestinal injury via ROS generation leading to mitochondrial dysfunction [43]. Many of the crucial events in apoptosis are centered on mitochondria, and that includes cyt. c release, changes in electron transport, loss of ΔΨ*m* and altered cellular oxidation-reduction. Nearly, all functions of mitochondria are associated with oxidative phosphorylation and energy coupling machinery located in the inner mitochondrial membrane. It consists of the ETC complexes I, II, III and IV, ATP synthase, ubiquinone, and cyt. c as electron transporters [26]. The ETC is considered a chief site of ROS generation, however, mitochondria are themselves vulnerable to oxidative damage, resulting in the impairment of respiratory enzyme activities, and thus affecting ΔΨ*m* and further production of ROS. Mitochondrial ROS generation is the net effect of ROS production at the ETC and their riddance by antioxidative enzymes. These two events rely greatly on the mitochondrial redox status, which is in turn dynamically influenced by various physiological and pathological settings [35]. The lipids, proteins, oxidative phosphorylation of enzymes, and mitochondrial DNA are all affected by ROS, thus ensuing cell death via apoptosis or necrosis [44]. Cyt. c oxidase is the terminal oxidase of the mitochondrial ETC, whose activity is also associated with apoptosis and has been reported to be in the reduced state during mitochondria-driven apoptosis. The structure and function of the enzyme is altered under different pathological conditions such as cancer, neurodegenerative diseases, CVDs, diabetes etc. It is thought that cyt. c oxidase dysfunction is most often associated with increased mitochondrial ROS production and cellular toxicity [45].

Cardiolipin oxidation is in recent times regarded as a significant factor not only in mitochondrial dysfunction, but also in the early stages of the mitochondrial apoptotic pathway [46]. Cardiolipin is an early target of ROS; oxidized cardiolipin flips from the inner to the outer mitochondrial membrane paving way for the formation of MPTP in the presence of Ca^2+^ released from ER and Golgi in response to stress, thus leading to leakage of cyt. c into the cytosol [47]. It has been reported that MTX mobilizes intracellular Ca^2+^ stores and that the their exhaustion might be responsible for the hepatotoxic and apoptotic effects of MTX [48]. From the results of the current study, it is clear that MTX induces apoptosis in platelets as evidenced by elevated activity of ETC enzymes such as NADH:ubiquitin-oxidoreductase causing increased ROS. It is also observed that MTX-induced phosphorylation of eIF2α causes ER stress, which in turn causes increased cytosolic Ca^2+^. Increased levels of ROS and Ca^2+^ further results in decreased ΔΨ*m*, cyt. c oxidase, augmented cardiolipin peroxidation as well as increased concentration of cytosolic cyt. c. These results are in line with a recent study by Kolli et al. [43], which demonstrated that MTX-induced mucositis is linked to the alteration of mitochondrial structure resulting in loss of function, and diminished activities of ETC components in enterocytes. The pro-apoptotic nature of MTX in platelets, was further confirmed by increased caspase-9 and -3 activities, PS externalization, protein tyrosine phosphorylation, as well as MTT and LDH assays. All the above-mentioned MTX-induced platelet apoptotic events were suppressed by NAC and more potently by NACA, underscoring the anti-apoptotic efficacy of these compounds. Though, MTX significantly promotes apoptosis in platelets, it neither affected various agonist-induced platelet aggregation nor on blood coagulation, suggesting that MTX does not activate platelets but rather triggers them to undergo apoptosis.

Investigation of the mechanism of MTX-induced platelet apoptosis apparently demonstrated the involvement of JNK signaling. MTX-induced ROS production is considered to be the most prominent mitochondrial inducer of JNK signaling in HeLa cells [49]. Activated JNK is reported to induce Bax, Bad and tBid leading to ΔΨ*m* dissipation and cyt. c release causing apoptosis in many nucleated cells [50]. JNK phosphorylation causes dephosphorylation of Bad (Ser136) and Bcl-2, and dissociates Bad from Bcl-XL or Bcl-2, resulting in elevated levels of Bad and Bcl-2 [51]. Here we demonstrate that MTX apparently induces phosphorylation of JNK, which in turn dephosphorylate Bad and elevate the levels of pro-apoptotic Bcl-2 family proteins, particularly Bax, Bad and tBid. This causes decreased ΔΨ*m* with cyt. c release and caspase activation in platelets leading to apoptosis. Use of specific inhibitor for JNK clearly demonstrates that phosphorylation of JNK is a key in the MTX-driven apoptosis in platelets. In addition, Mito-TEMPO, mitochondria-targeted ROS antagonist completely abolished MTX-induced ROS production along with phosphorylation of JNK, and downstream PS externalization indicating that mitochondria are the major sources for ROS production in platelets and is responsible for MTX-induced platelet toxicity. NAC/NACA treatment effectively suppressed JNK-mediated activation of Bcl-2 family proteins by MTX as well as the subsequent apoptotic events. NAC being a precursor of cellular L-cysteine and reduced GSH, also scavenges free radicals by interacting with ROS [52]. In the current scenario, NAC is assumed to scavenge free radicals generated by impaired electron transport during MTX treatment and also increase cellular reduced glutathione content. The obtained results clearly suggest that mitochondrial ROS are the key mediators of MTX-induced apoptosis. Thereby, NAC and its derivative NACA intercede at the root cause and regulate MTX-induced apoptosis. Finally, it is demonstrated that MTX promotes apoptosis in platelets by activation of ROS-JNK-mediated mitochondrial damage and NAC/NACA protects platelets by increasing reducing equivalent levels and scavenging ROS.

Besides, when platelets are activated or undergo apoptosis, the normal asymmetrical distribution of lipids between the inner and outer leaflets of a plasma membrane is disturbed, resulting in blebbing of plasma membrane and shedding of procoagulant MPs. Platelet-derived MPs (PMPs) are a pool of bioactive effectors and are able to act as intercellular messengers, regulate inflammation, and influence vascular functions, apoptosis and cellular cross talk. PMPs play a significant role in the pathogenesis of RA, cancer metastasis and other vascular diseases [53].

## Conclusion

Despite the therapeutic potential of MTX as anti-rheumatic and anti-cancer drug, recent reports demonstrate that it leads to an oxidative catastrophe. MTX, being a pro-oxidant, aggravates endogenous ROS production at the cellular level and provokes oxidative damage to liver, kidney, and even to central nervous system. Notable incidences of thrombocytopenia in MTX-treated RA and cancer patients convey that MTX in the circulation along with the augmented levels of cellular ROS that have infiltrated into circulation also affect blood components. Here, we appraise the pro-apoptotic action of MTX on platelets and its inhibition by NAC, a clinically approved potent thiol antioxidant and its derivative NACA. MTX remarkably accelerates ROS-mediated oxidative stress in platelets triggering apoptotic events via JNK-mediated mitochondrial damage. The treatment of NAC/NACA efficiently restrains MTX-induced oxidative stress and apoptosis of platelets. The present study for the first time reports the mechanism of action of MTX on platelets and establishes a connection with thrombocytopenia in MTX-treated RA/cancer patients. The results of the present study pose apprehensions in the involvement of pro-oxidants in therapeutic regimen of human ailments, as it might cause added stress to the already ailing system and may lead to severe secondary complications. It may even lead to resurgence of the disease owing to MP generation. The unwarranted levels of MPs in circulation might also result in the manifestation of yet another disease and this vicious cycle seems to go on and on. Furthermore, our study also underpins the use of clinically potent antioxidants as auxiliary to MTX treatment regime considering the significant role played by the latter in cancer and RA treatment.

## Supporting Information

S1 Fig Effect of MTX on platelet aggregation induced by (A) Collagen (B) ADP (C) Epinephrine. [Blue trace- Collagen/ADP/Epinephrine alone, black trace- MTX 10 μM, brown trace- MTX 25 μM, green trace- MTX 50 μM] **(D)** Graphical representation of the data showing percentage platelet aggregation. Values are presented as mean ± SEM (n = 5).
(TIF)
