# Supplementary figures and images for: Methotrexate Promotes Platelet Apoptosis via JNK-Mediated Mitochondrial Damage: Alleviation by N-Acetylcysteine and N-Acetylcysteine Amide
Source: PLoS One. 2015 Jun 17;10(6):e0127558. doi: 10.1371/journal.pone.0127558 (PMC4471342; doi:10.1371/journal.pone.0127558)

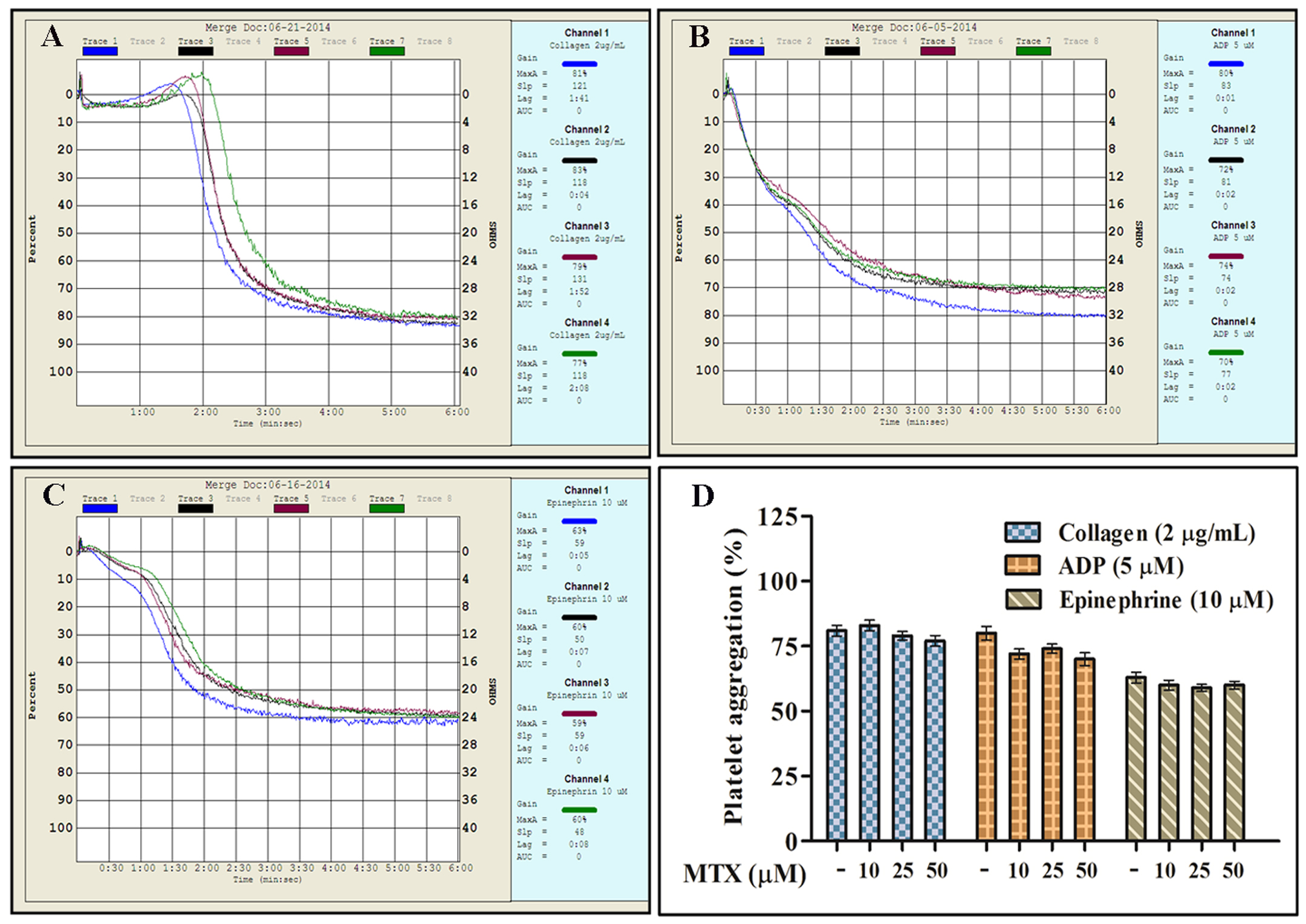

Supplement: S1 Fig — [Blue trace- Collagen/ADP/Epinephrine alone, black trace- MTX 10 μM, brown trace- MTX 25 μM, green trace- MTX 50 μM] (D) Graphical representation of the data showing percentage platelet aggregation. Values are presented as mean ± SEM (n = 5). (TIF) [file pone.0127558.s001.tif]
